# Supplementary material for: The effect of voluntary disclosure on financial performance: Empirical study on manufacturing industry in Indonesia
Source: PLoS One. 2023 Jun 2;18(6):e0285720. doi: 10.1371/journal.pone.0285720 (PMC10237478; doi:10.1371/journal.pone.0285720)
Supplement: Attachment — Submitted filename: Response to Reviewers Letter-250423.docx [file pone.0285720.s001.docx]

**Response to Reviewers’**

Many thanks to reviewer 1 for valuable comment

1) Reviewer 1 : The title must be complete by adding ‘in Indonesia’ to make it complete

Authors : we have done revision please see page 1

2) Reviewer 1 : The abstract must have a conclusion and recommendations of the study

Authors : we have done revision please see page 1

3) Reviewer 1 : The last paragraph of your introduction section must end up by giving clues about the organisation of the paper. Revise.

Authors : we have done revision please see page 3

4) Reviewer 1 : The literature section must capture empirical literature on CSR Strategies, CSR Disclosures and CSR initiatives and activities

Authors : we have done revision please see page 3-5

5) Reviewer 1 : How the data were extracted from the Annual Reports must be captured for replication purposes. That is the procedure. The Data Description and the measurement of the variables must be reported.
 Authors : we have done revision please see page 9-12

6) Reviewer 1 : The result section must report the data for each company that makes up the 13 companies as the study sample. This will be insightful for readership and analysis. Putting all the 13 companies as an entity will not reveal the undertones of the individual company’s

Authors : we have done revision please see page 18-

7) Reviewer 1 : • These references must be deleted from the reference list as they did not appear in the text. Reference numbers 5, 9, 14, 15 and 16.

Authors : we have done revision please see page

8) Reviewer 1 : Incomplete reference sources, either with page numbers for the journals or the absence of the location of the publisher for books. Reference numbers 4, 6, 7, 10, 11, 17, 18, 24 and 25

Authors : we have done revision please see page 26-27

9) Reviewer 1 : The author(s) must do well to improve the quality of English in the paper

Authors : we have done revision please see page 1-28

**Responses to Reviewer**

Many thanks to reviewer 2 for valuable comment

Abstract
1. Reviewer : The first line disappointed me; rather, it should be like this…. "The obedience of the manufacturing sector industry to manage natural resources from the environment still needs to be higher.

Authors : we have done revision please see page 1

2. Reviewer : There is a need to improve the style of writing.

Authors : we have done revision please see page 1

3. Reviewer : Without highlighting the purpose of the study, the authors have straight forwarded moved to the research methodology. I am not happy with the English part of this manuscript in the abstract section.
Authors : we have done revision please see page 1

Introduction
1. Reviewer : Background of the study is missing. I cannot see any outline or an overview of the topic

Authors : we have done revision please see page 1

2. Reviewer : What is the source of this? According to the Ministry of Environment and Forestry (KLHK), the compliance of the manufacturing sector industry in managing natural resources from the environment is still low. Until 2019, the number of companies that registered themselves to be assessed for compliance was quite low, only reaching 597 companies or 29.15 per cent."

Authors : we have done revision please see page 2

3. Reviewer : I do not understand this” In this research does not expand and is more focused on the research objectives that have been described, the research limitations are…."

Authors : we have done revision please see page 22-23

4. Conducted on manufacturing industry companies: can you please define why you have selected manufacturing industry?

Authors : we have done revision please see page 3

5. The author said “manufacturing industry companies” is this correct way of writing?

Authors : we have done revision please see page 1-25

Literature review
1. Reviewer : It could be more satisfactory. Please focus on the research gap .. Strengthen the argument of your study by referring to a larger body of scientific literature, and clearly outline what is missing in the literature and what gap you wish to fill in. Why is the focus only on old theories? Kindly add relevant latest literature since CSR is the talk of the town. It is a part of financial disclosure.

Authors : we have done revision please see page 1-3

2. Reviewer : What are the selection criteria for this 91 indicator? Please clarify.

Authors : The 91 indicators used in this study is based on GRI G4 guidelines. we have done revision please see page 5

Research methodology
1. Reviewer : Please elaborate on how and why purposive sampling.

Authors : The reason for using this purposive sampling technique is because it is in accordance with this research, namely using quantitative research, and refer to previous research and studies that do not generalize (Uma Sekaran, 2016). we have done revision please see page 10

2. Reviewer : What was the population size here?

Authors : namely 45 companies, we have done revision please see page 10

3. Reviewer : In the Data Collection Method, who were the respondents

Authors : in this study we use secondary data, namely data collected by www.idx.co.id, so we don't use quesioner fill by respondent, you can see in page 10-12

Conclusion
1. Reviewer : I can’t see much difference between discussion and conclusion part. Actually the author has written a small portion of the analysis and discussion part in the conclusion.

Authors : we have done revision please see page 17-23

Kindly rewrite the entire conclusion section. There is a need for the division of discussion and conclusion into two separate sections, with the conclusion section simply needing to give the most critical information in points so that readers can quickly find what they want from the publication. The discussion section might need to add more subheadings, such as a discussion of the findings, main contributions, policy recommendations, limitations, and future work of this study. The manuscript's logic would substantially increase if the authors were willing to modify it.

Authors : we have done revision please see page 17-23

References
1. Reviewer : Many mistakes in references like volume nos and page nos etc. are missing, for example: 23-25, 3, 5, 4, 7, 8. there is a need to go through all the references carefully. Please check all the references.

Authors : we have done revision please see page 23-27

2. Reviewer : In text style of citation is also not properly followed. Mistakes at many places and need to add more citations.

Authors : we have done revision please see page 1-27

Best Regards,

Dr. Meiryani
